# The *SbTDR* gene is required for anther development and male fertility in *Sorghum bicolor*

**DOI:** 10.1007/s00497-026-00546-4

**Published:** 2026-08-25

**Authors:** Ashley R. Smith, Jian Huang, Uyiosasere D. Aigbe, Xia Wan, Junping Chen, Zhanguo Xin, Dazhong Zhao

**Affiliations:** 1 Department of Biological Sciences, University of Wisconsin-Milwaukee, Lapham Hall, S181 3209 N. Maryland Ave, Milwaukee, WI 53211 USA; 2 Plant Stress and Germplasm Development Unit, USDA-ARS, Lubbock, TX 79415 USA

**Keywords:** Anther development, Tapetum, Male sterility, the TDR bHLH transcription factor, Hybrid breeding, Sorghum

## Abstract

Anthers are essential in plant reproduction and are also crucial in agriculture, because hybrid seed production typically relies on the manipulation of male fertility. Sorghum (*Sorghum bicolor* L. Moench) is the fifth most produced grain crop in the world. However, relatively little is known about the genetic mechanisms that regulate anther development and male fertility in sorghum. Here, we report the morphological characterization of anther development in wild-type sorghum BTx623 and the *Sorghum bicolor tapetal degeneration retardation* (*Sbtdr*) mutant, as well as identification of the causal gene *SbTDR*. Our results showed that the *Sbtdr* mutant formed small, white anthers that failed to produce pollen grains. We classified sorghum anther development into 13 main stages based on major cellular landmarks observed in semi-thin sections of anthers. Our analysis demonstrated that the *Sbtdr* mutant is defective in tapetal cell degeneration. Moreover, in situ hybridization revealed that the *SbTDR* gene, which encodes a bHLH transcription factor, is primarily expressed in tapetal cells at the microspore stage. Our discoveries highlight the importance of the SbTDR bHLH transcription factor in controlling tapetal cell differentiation and male sterility, which may help develop novel hybrid breeding systems in sorghum.

**Supplementary Information:**

The online version contains supplementary material available at 10.1007/s00497-026-00546-4.

## Key Message

The *SbTDR* gene, which encodes a bHLH transcription factor, regulates tapetum degeneration and male sterility, potentially enabling hybrid breeding in sorghum.

**Supplementary Information:**

The online version contains supplementary material available at 10.1007/s00497-026-00546-4.

## Introduction

The stamen is the male reproductive organ of flowering plants, consisting of an anther where the male gametophyte (pollen) develops and a filament that anchors the anther to the flower. A typical anther has four lobes (microsporangia) (Feng et al. 2013; Goldberg et al. 1993; Walbot and Egger 2016; Zhao 2009). Within each locule, the central reproductive microsporocytes (pollen mother cells) are surrounded by four concentrically organized layers of somatic cells: the epidermis, endothecium, middle layer, and tapetum (from outside to inside). Microsporocytes give rise to pollen via meiosis, while the surrounding somatic cell layers, particularly the tapetum, are required for the normal development and release of pollen. Abnormal anther development causes partial or complete male sterility; therefore, the anther is essential to plants not only for producing offspring, but also for supporting agriculture.

Tapetum, comprising a monolayer or multilayers of endopolyploid cells, is required for pollen development (Goldberg et al. 1993; Scott et al. 2004; Walbot and Egger 2016). Generally, tapetal cells secrete enzymes (e.g., β-1,3-glucanase) required for releasing haploid microspores from tetrads by breaking down the callose surrounding the meiotic tetrads (Clement and Pacini 2001; Hsieh and Huang 2007; Ishiguro et al. 2010; Pacini et al. 1985; Parish 2010), and provide energy and materials for pollen development (Huang et al. 2017; Wang et al. 2003; Wu et al. 1997). With endoreduplication mature tapetal cells contain multiple nuclei and specialized non-photosynthetic plastids (elaioplasts) and tapetosomes, which provide lipids, proteins, polysaccharides and sporopollenin necessary for pollen wall formation (Hernandez-Pinzon et al. 1999; Hsieh and Huang 2007). Moreover, tapetal cells degenerate via programmed cell death (PCD), and depending on the plant species, materials such as tryphine and pollenkitt are deposited to form the pollen coat (Hsieh and Huang 2007; Li et al. 2011). Plants lacking a functional tapetum fail to produce pollen grains and consequently are male sterile (Mariani et al. 1990; Zhao et al. 2002; Huang et al. 2016a). Moreover, precocious or delayed tapetum degeneration impairs pollen development and results in male sterility (Fu et al. 2014; Niu et al. 2013; Parish 2010; Zhang et al. 2006). Tapetal cells are highly stress-sensitive, thus, stress-induced male sterility and its associated seed yield losses are often attributed to aberrant tapetum development and/or abnormal programmed cell death (Begcy et al. 2019; Chaturvedi et al. 2021; De Storme and Geelen 2014; Malik and Zhao 2022; Parish et al. 2012; Smith et al. 2023; Smith and Zhao 2016).

Many pathways are involved in controlling tapetum development and function. For example, the EMS1 (EXCESS MICROSPOROCYTES1) Leucine-Rich Repeat Receptor-Like Kinase (LRR-RLK)-linked signaling pathway, including the EMS1 receptor kinase (Zhao et al. 2002), the small protein ligand TPD1 (TAPETUM DETERMINANT1) (Huang et al. 2016b, c; Jia et al. 2008), co-receptor SERK1/2 (SOMATIC EMBRYOGENESIS RECEPTOR-LIKE KINASE1/2) (Li et al. 2017), and downstream effector β-CA (β-Carbonic Anhydrase) (Huang et al. 2017), plays a fundamental role in tapetal cell differentiation during early anther development in *Arabidopsis*. Mutations in genes encoding these signaling proteins lead to the ablated or aberrant tapetum, and hence, male sterility. Analyses of TPD1 and EMS1 orthologues suggest a conserved role for TPD1-EMS1 signaling in anther cell differentiation in crop plants, including rice and maize (Hong et al. 2012; Kelliher and Walbot 2012; Nonomura et al. 2003; van der Linde et al. 2018; Wang et al. 2012; Zhao et al. 2008).

Transcription factors in the bHLH (basic Helix-Loop-Helix) family, which contains more than 150 members in *Arabidopsis* and rice, are critical for tapetal cell differentiation and degeneration (Ortolan et al. 2024; Pires and Dolan 2010). Loss-of-functions of *DYT1* (*DYSFUNCTIONAL TAPETUM1*) (Zhang et al. 2006) and *bHLH010*/*bHLH089*/*bHLH091* (Zhu et al. 2015) genes in *Arabidopsis* and their rice orthologous genes *UDT1* (*UNDEVELOPED TAPETUM1*)/*bHLH164* (Jung et al. 2005), *TDR* (*TAPETUM DEGENERATION RETARDATION*)/*bHLH5* (Li et al. 2006; Zhang et al. 2008), *TIP2* (*TDR INTERACTING PROTEIN2*)/*bHLH142* (Fu et al. 2014), and *EAT1* (*ETERNAL TAPETUM1*)/*DTD* (*DELAYED TAPETUM DEGENERATION*)/*bHLH141* (Ji et al. 2013; Niu et al. 2013) results in the aberrant formation and degeneration of tapetal cells. In *Arabidopsis*, the formation of heterodimer between DYT1 and bHLH010/bHLH089/bHLH091 facilitates the nuclear localization of DYT1 in tapetal cells (Zhu et al. 2015). In rice, the complicated interactions among UDT1, TDR, TIP2, and EAT1/DTD are required for normal tapetum development. UDT1 activates the *TIP2* transcription, while the TIP2-TDR heterodimer directly regulates expression of *EAT1* and *TDR via* a feed-forward regulatory mechanism (Fu et al. 2014; Ko et al. 2014). EAT1 can also dimerize with TDR, which competes with the activity of the TIP2-TDR heterodimer. Additionally, EAT1 directly activates expression of two aspartic protease genes *AP25* and *AP37*, which might induce tapetal programmed cell death (Ko et al. 2014; Niu et al. 2013). Moreover, EAT1, via the possible interaction with TIP2 and UDT1, promotes biogenesis of 24-nt phasiRNAs (24-nucleotides phased secondary small interfering RNA) (Ono et al. 2018). In maize, bHLH122 (the EAT1 orthologue), MS23 (MALE STERILE23, the TIP2 orthologue), MS32 (the UDT1 orthologue), and bHLH51 (the TDR orthologue) control tapetal cell differentiation and function via a similar regulation mechanism as described above (Moon et al. 2013; Nan et al. 2017).

Sorghum [*Sorghum bicolor* (L.) Moench] is the fifth most produced grain crop globally behind wheat, maize, rice, and barley. As a C4 crop with excellent tolerance to drought and high temperature stresses, sorghum is emerging as a promising food, feed, and fuel crop adapted to lower-input conditions than maize [*Zea mays* L.] and rice [*Oryza sativa* L.] (Jiao et al. 2016, 2018; Smith et al. 2023; Xin et al. 2017). In sorghum, previous studies have reported nine nuclear male sterility (NMS) mutants designated *ms1* through *ms9* (Chen et al. 2019; Xin et al. 2017). Both *ms8* and *ms9* mutants are defective in tapetal cell degeneration, which results in the failure of pollen production. The *MS9* gene encodes a plant homeotic domain (PHD) finger transcription factor (Chen et al. 2019). Anther development is widely studied in many cereal grains and model organisms, including rice, maize, and *Arabidopsis*; however, less is understood about anther cell differentiation at both morphological and molecular levels in sorghum (Chen et al. 2019; Laza et al. 2022; Smith et al. 2023; Xin et al. 2017).

In this study, we report the isolation and characterization of the *Sbtdr* male sterile mutant in sorghum, whose causal gene is orthologous to the rice *TDR* gene. *Sbtdr* mutant plants exhibit complete male sterility, producing no viable pollen. We established a 13-stage developmental series for sorghum anthers based on semi-thin section analysis, which serves as a valuable framework for characterizing anther defects in sorghum male sterile mutants. Our analysis revealed that *Sbtdr* anthers are defective in timely tapetal degeneration, which leads to microspore abortion and failure of pollen production. The *SbTDR* gene encodes a bHLH transcription factor and is predominantly expressed in tapetal cells at the microspore stage. Our findings demonstrate that the *SbTDR* gene plays a key role in pollen development by controlling tapetal degeneration in sorghum. Moreover, our results have potential applications to developing novel hybrid breeding systems in sorghum.

## Materials and methods

### Sorghum lines and growing conditions

*Sorghum bicolor* (L.) Moench BTx623 (wild type), *Sbtdr*-*1* (*ARS*-*67*), and *Sbtdr*-*2* (*ARS*-*84*) seeds were grown in SUN GRO Metro-Mix 360 soil. The plants were grown under greenhouse conditions of 28/23˚C (day/night), with 10 h of light (8am-6pm), 14 h of dark (6pm-8am), and a relative humidity of 50%. Plants were watered daily and fertilized weekly using a Young Mixer-Proportioner Injector at a rate of 60:1, with Sprint 330 and Peterson’s Water Soluble General Purpose 20-20-20 fertilizer (250 ppm N and 20 ppm Fe).

### Generation and identification of mutants in the SbTDR gene

*Sbtdr*-*1* (ARS-67) and *Sbtdr*-*2* (ARS-84) mutants were identified through a screening of the sorghum mutant library established as described previously (Jiao et al. 2016, 2017; Xin et al. 2008). Briefly, seeds of the sorghum inbred line BTx623 (100 g, approximately 3,300 seeds) were treated with ethyl methanesulfonate (EMS) at concentrations ranging from 0.1% to 0.3% (v/v). The mutagenized seeds (M1) were planted at the Research Farm of the Plant Stress and Germplasm Development Research Unit, USDA-ARS, in Lubbock, Texas, USA, to generate M2 seeds, which were subsequently planted to produce M3 seeds. A mutant library was created in the M3 generation.

Mutations in the *TAPETAL DEGENERATION RETARDATION* (*TDR*) gene cause male sterility in rice (Li et al. 2006; Zhang et al. 2008). To identify sorghum *tdr* mutants, we searched our sequenced sorghum mutant library using the *SbTDR* gene (Sorbi_3004G017500) sequence. We identified two mutant lines, originally designated *ARS67* and *ARS84*, both of which harbor mutations in the *SbTDR* gene and segregated male sterile plants. Genetic complementation analysis showed that male sterility was observed in the F1 generation, suggesting that mutations in the same *SbTDR* gene are responsible for the phenotype.

To verify the mutations, the *SbTDR* genomic fragments were amplified by PCR from *Sbtdr-1* and *Sbtdr-2* mutants using primers in Table S1 and then sequenced by GENEWIZ. These mutant sequences were aligned to the WT sequence downloaded from Phytozome (https://phytozome.jgi.doe.gov/pz/portal.html) to determine the presence and location of the mutations.

### Examination of male fertility

The male fertility was tested by Alexander pollen staining as described previously (Alexander 1969; Smith et al. 2023; Xin et al. 2017). Briefly, the spikelets with anthers just before dehiscence were collected and fixed in Carnoy’s fixative (60% ethanol, 30% chloroform, and 10% glacial acetic acid). Anthers were dissected out and transferred through 70%, 50%, and 30% ethanol, followed by distilled water (1 h in each exchange). The anthers were then incubated in the staining buffer (ethanol 95%, 10 ml; malachite green, 10 mg; acid fuchsin, 50 mg; orange G, 5 mg; phenol, 5 g; glacial acetic acid, 2 ml; glycerol, 25 ml; and distilled water 50 ml) at 50 °C for 48 h. Anthers were mounted on the glass slide for observation using an Olympus BX51 microscope equipped with an Olympus DP 70 digital camera (Olympus, Center Valley, PA, USA). The anthers were evaluated as fertile if they contained large red pollen grains, while the absence of pollen grains in anthers indicated male sterility.

### Examination of female fertility

The female fertility was examined as described in our previous study (Xin et al. 2017). After panicles emerged, the *Sbtdr* mutant plants were determined by anther phenotype. The exposed portions of the panicles of BTx623 and *Sbtdr* plants were cut and bagged. In the following morning, the freshly opened spikelets were manually pollinated with the BTx623 pollen and then bagged again. Two and three days after pollination, spikelets were collected, and ovaries were dissected out. Then, the enlargement of the ovaries was observed and imaged with the Olympus SZX7 dissection microscope equipped with an Olympus DP 70 digital camera (Olympus, Center Valley, PA, USA).

### Semi-thin sectioning and imaging

Semi-thin sectioning was carried out as described in our previous studies (Huang et al. 2017; Smith et al. 2023; Xin et al. 2017). Briefly, panicles were harvested from 6- to 10-week old BTx623 and *Sbtdr* plants and the floral tissues were fixed in Trump’s 4:1 primary fixative [1% glutaraldehyde, 4% paraformaldehyde, 0.1 M HEPES, and 0.02% Triton X-100 (pH 7.2)](McDowell and Trump 1976). Floral tissues were vacuum infiltrated in the primary fixative for 10–30 min, then left at room temperature overnight and stored at 4 °C until use. Following the post-fixation in 1% of osmium tetroxide overnight at room temperature, floral tissues were dehydrated through an acetone series (10% increments, 1 h each exchange) and then infiltrated in 20%, 40%, 60%, and 80% of Spurr’s resin (3 h each exchange). Floral tissues were finally embedded in molds filled with 100% resin. Semi-thin sections of 0.5 μm were cut on an Ultracut E ultramicrotome (Reichert-Jung). The sections were stained with 0.05% of Toluidine Blue O and rinsed with water. Anther sections were observed and imaged on an Olympus Bx51 microscope equipped with an Olympus DP70 digital camera (Olympus, Center Valley, PA, USA).

### Phylogenetic analysis

Protein sequences of *Sb*TDR and orthologous proteins from representative monocot and dicot species were retrieved from UniProt. The sequences were aligned with MAFFT in Geneious v9.0.2 (Kearse et al. 2012). The best-fit model was selected by ModelFinder, and a maximum likelihood tree was reconstructed in IQ-TREE v.1.6.12 with 1,000 ultrafast bootstrap replicates (Nguyen et al. 2015).

### RT-PCR and RNA in situ hybridization

Samples were collected from seedlings and spikelets spanning developmental stages from pre-microsporocyte (or PMC: pollen mother cell) to pollen maturation. Fresh floral tissues were dissected, and anthers from pre-microsporocyte through the pollen stage were removed and processed separately. All tissues were preserved in RNAlater at -20 °C until sufficient materials were accumulated. Total RNA was extracted using the RNeasy plant mini kit (Qiagen) (Zhao et al. 2017), followed by cDNA synthesis using Qiagen’s QuantiTect Reverse Transcription Kit according to the manufacturer’s instructions. Expression of the *ACTIN* gene was used to calibrate the cDNA quality prior to performing RT-PCR for *SbTDR*. Primer sequences used for amplification are listed in Table S1.

RNA in situ hybridization was performed using anthers from BTx623 as described previously (Huang et al. 2016c, 2022; Klocko et al. 2016; Zhao et al. 2002). The SP6/T7 DIG RNA Labeling Kit (Roche) was used to generate *SbTDR* sense and antisense probes. Primers for PCR and RNA in situ hybridization were listed in Supplemental Table S1.

## Results

### Sbtdr is a male sterile mutant in sorghum

**Fig. 1:**
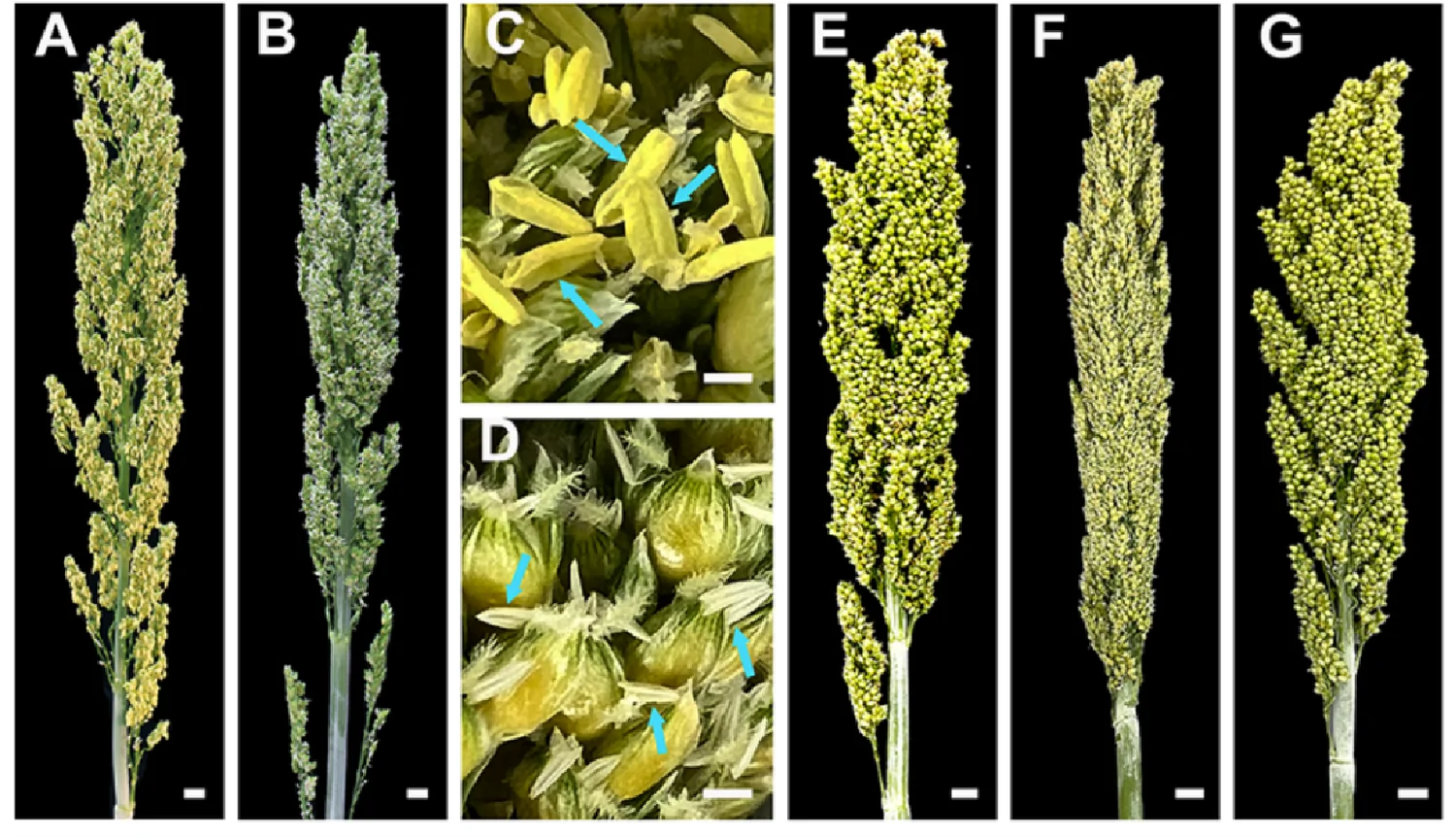
The *Sbtdr* mutant is male sterile. (**A**) A BTx623 panicle exhibiting yellow anthers at anthesis. (**B**) A *Sbtdr-1* panicle displaying white anthers at anthesis. (**C**) A portion of BTx623 panicle showing yellow anthers (arrows). (**D**) A portion of *Sbtdr-1* panicle exhibiting white anthers (arrows). (**E**) A self-pollinated BTx623 panicle displaying normal seed set. (**F**) A bagged *Sbtdr-1* panicle showing the absence of seed set. (**G**) The seed set of a cross-pollinated *Sbtdr-1* panicle is comparable to that of BTx623. Scale bars: 1 cm (A, B, E-G) and 1 mm (C, D)

**Fig. 2:**
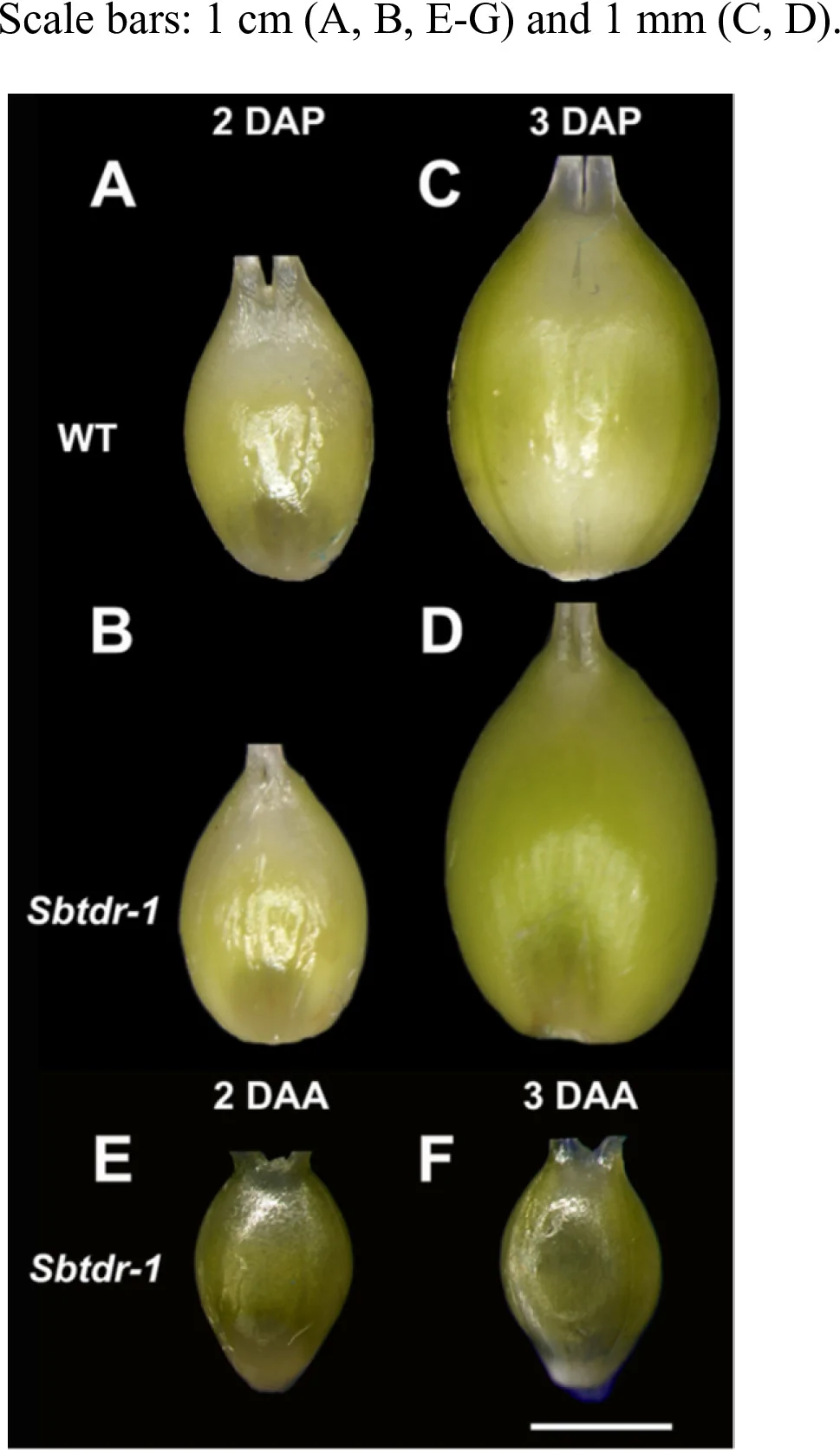
Female fertility is normal in the *Sbtdr* mutant. (**A**, **B**) Ovaries of BTx623 (**A**) and *Sbtdr-1* (**B**) plants 2 days after pollination (DAP) with BTx623 pollen are the same size. (**C**, **D**) Ovaries of BTx623 (**C**) and *Sbtdr-1* (**D**) plants 3 DAP with BTx623 pollen exhibiting comparable enlargement. (**E**, **F**) Ovaries of unpollinated *Sbtdr-1* 2 days after anthesis (DAA, **E**) and 3 DAA (**F**) showing no increase in size. Scale bars: 1 mm

To identify genes governing male sterility in sorghum. We screened for mutations in genes involved in anther development in *Arabidopsis* and rice using a sorghum mutant library generated by EMS mutagenesis of seeds from the elite inbred line BTx623 (Jiao et al. 2016, 2017; Xin et al. 2008). We found two mutant lines designated *ARS-67* and *ARS-84* that carried point mutations in the sorghum orthologue of the rice *TDR* gene (gene ID: Sorbi_3004G017500). Each line segregated mutants that were unable to produce seeds and pollen, we then renamed them *Sbtdr-1* (*ARS-67*) and *Sbtdr-2* (*ARS-84*). In this study, we focused on characterizing phenotypes of the *Sbtdr-1* mutant. During anthesis, wild-type BTx623 panicles produced yellow anthers (Fig. 1A, C), whereas anthers in *Sbtdr-1* panicles were smaller and white (Fig. 1B, D). Unlike BTx623 panicles with a full set of seeds (Fig. 1E), seed sets were not observed in *Sbtdr-1* panicles when bagged to prevent outcrossing (Fig. 1F). However, seed production was fully restored when *Sbtdr-1* mutant plants were pollinated with BTx623 pollen (Fig. 1G), and approximately 80 F1 plants were fully fertile, suggesting that *SbTDR* is a recessive gene and that a single wild-type *SbTDR* allele is sufficient to restore fertility. The F2 plants segregated into 36 male sterile and 112 fertile plants, consistent with the expected 1:3 ratio (mutant: wild type) for a single recessive mutation. To assess female fertility, ovary enlargement was monitored following manual pollination of *Sbtdr-1* plants. In both BTx623 (Fig. 2A, C) and *Sbtdr-1* mutant (Fig. 2B, D) plants, ovaries enlarged normally post-pollination, showing no observable differences in female fertility 2 and 3 days after pollination (DAP). By contrast, the ovaries of unpollinated *Sbtdr-1* plants failed to enlarge (Fig. 2E, F). Collectively, these results indicate that *Sbtdr-1* is a male sterile mutant.

### The defects in tapetum degeneration cause the failure of pollen production in Sbtdr mutant plants

**Fig. 3:**
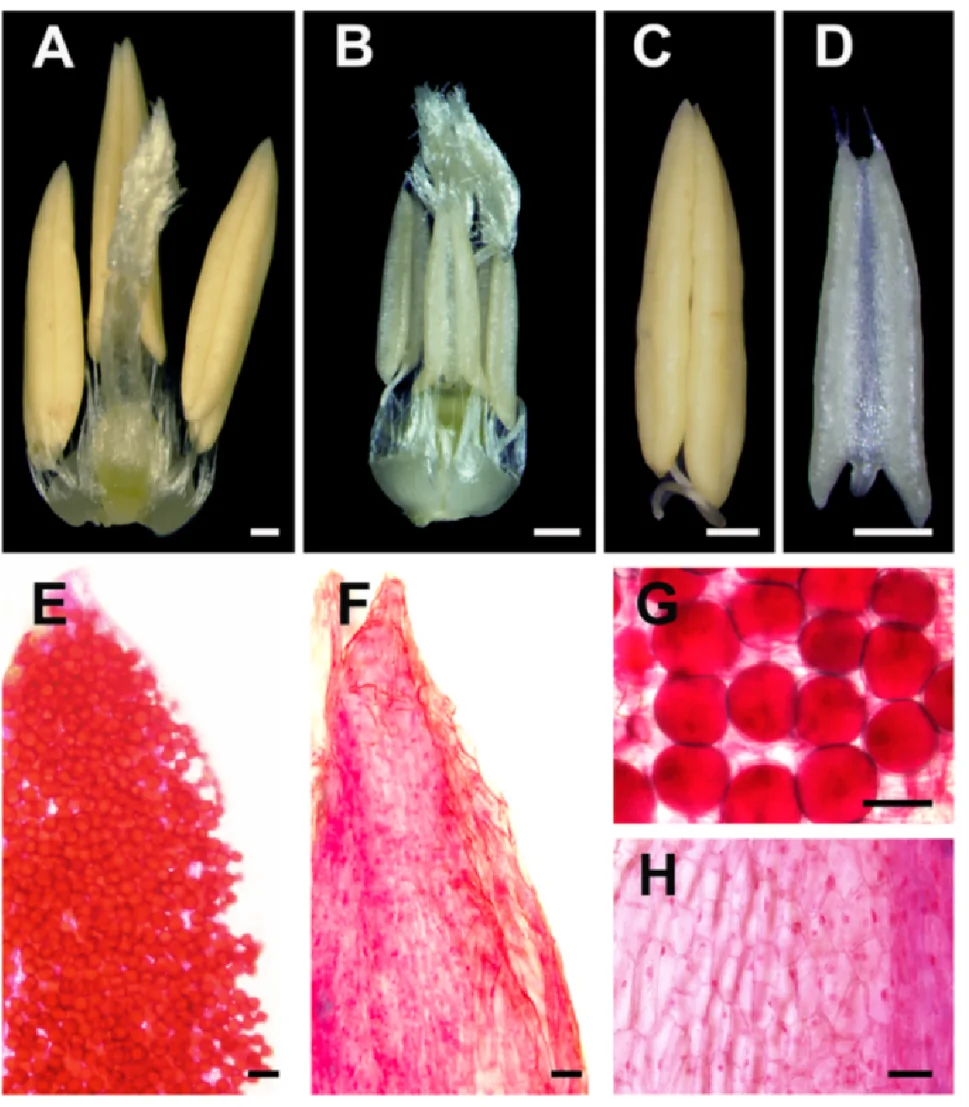
*Sbtdr* anthers fail to produce pollen grains. (**A**, **B**) The BTx623 sessile spikelet showing three yellow mature anthers and stigmas (**A**), while the *Sbtdr-1* sessile spikelet exhibiting three pale anthers and stigmas (**B**). Other floral organs were removed. (**C**, **D**) A single yellow BTx623 anther (**C**) and a pale *Sbtdr-1* anther (**D**). (**E**, **G**) Part of a BTx623 anther (**E**) and a high-magnification view (**G**) displaying round pollen grains in the anther locule. (**F**, **H**) Part of an *Sbtdr-1* anther (**F**) and a high-magnification view (**H**) showing the absence of pollen grains in the anther locule. Scale bars: 0.5 mm (A-D), 50 μm (E, F), and 25 μm (G, H)

To investigate the causes of male sterility in the *Sbtdr-1* mutant, we assessed pollen viability by Alexander pollen staining (Alexander 1969; Smith et al. 2023; Xin et al. 2017). BTx623 sessile spikelets have three yellow anthers (Fig. 3A, C). In contrast, *Sbtdr-1* sessile spikelets bear three small, white anthers (Fig. 3B, D), indicating a failure to produce pollen. Alexander staining revealed abundant, round, red-stained viable pollen grains in the BTx623 anther locule (Fig. 3E, G). Conversely, no pollen grains are present in the *Sbtdr-1* mutant anther locule (Fig. 3F, H), confirming that the *Sbtdr-1* mutant is incapable of pollen production.

**Fig. 4:**
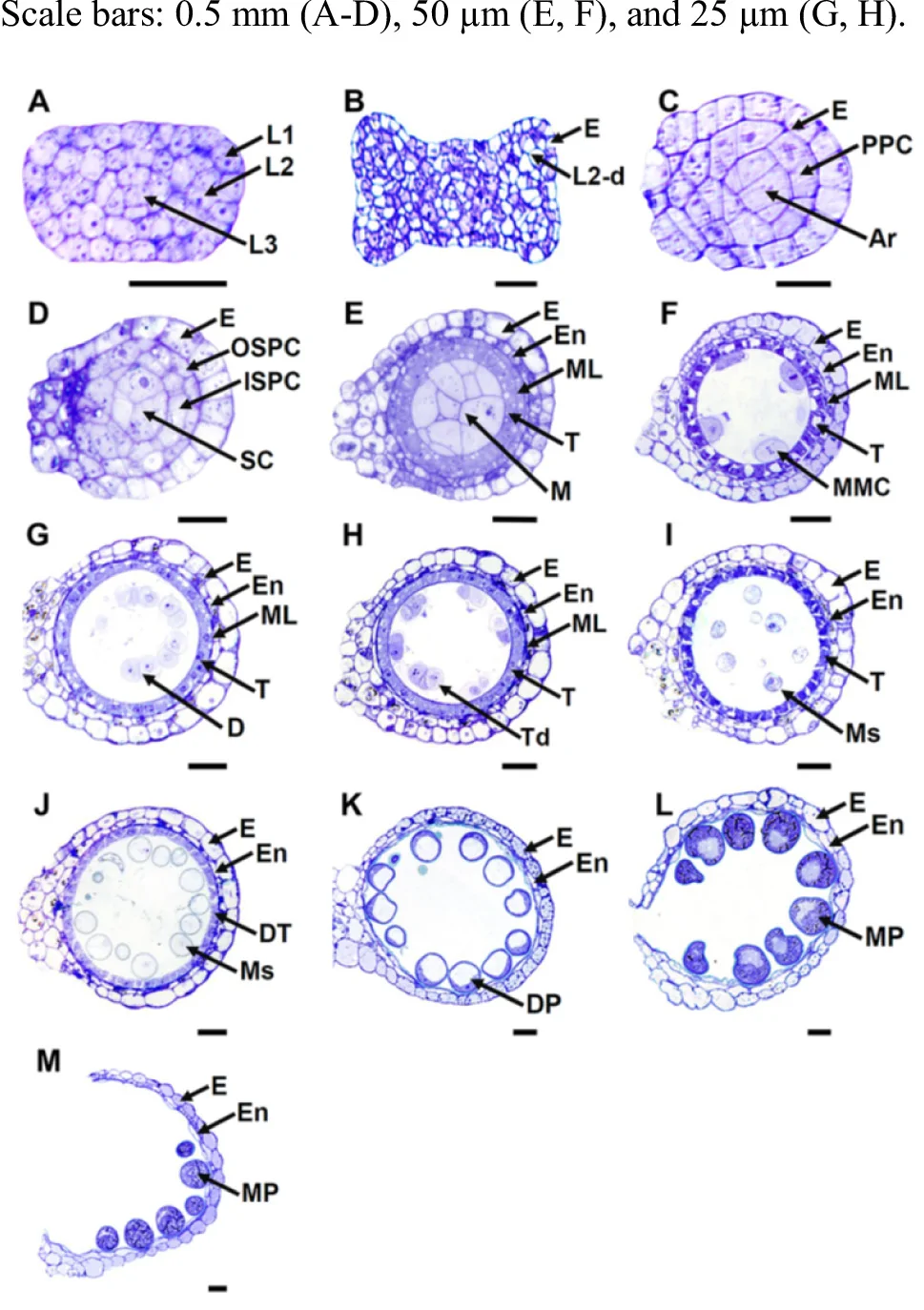
Defining stages of anther cell differentiation in sorghum by semi-thin section analysis. Image (**A**): an anther primordium, image (**B**): an entire anther, images (**C**) to (**M**): individual anther lobes. (**A**) Stage 1: An anther primordium consists of three layers (L): (L1), L2, and L3. (**B**) Stage 2: L2 gives rise to L2-derived cells (L2-d), and four anther lobes are visible. (**C**) Stage 3: Archesporial (Ar) cells and primary parietal cells (PPC) differentiate from L2-d cells. (**D**) Stage 4: PPCs divide to form outer and inner secondary parietal cells (OSPC and ISPC) formed from PPCs. Ar cells give rise to Sporogenous cells (SC). (**E**) Stage 5: Endothecium (En) is derived from OSPCs. The middle layer (ML) and tapetum (T) arise from ISPCs. Microsporocytes (M or pollen mother cells) appear. (**F**) Stage 6: Microsporocytes enter meiosis and are visible as isolated male meiotic cells (MMC). The tapetal cells become vacuolated. (**G**) Stage 7: The first meiotic division is completed, forming dyads (D). (**H**) Stage 8: The second meiotic division is completed, forming tetrads (TD). The tapetal cells become less vacuolated. (**I**) Stage 9: Individual microspores (MS) are released from tetrads following the degradation of the surrounding callose wall. The middle layer is no longer visible. (**J**) Stage 10: Microspores generate an exine wall and become vacuolated. Degeneration of tapetal cells initiates. (**K**) Stage 11: Pollen wall formation progresses, and tapetal cells are degenerated. (**L**) Stage 12: Pollen grains mature, and septum breakage occurs. (**M**) Stage 13: The anther dehisces, and mature pollen is released from the anther locule. Ar: Archesporial cells, D: Dyad, DP: Developing Pollen, DT: Degenerating Tapetum (Tapetal cells), E: Epidermis, En: Endothecium, L: Layer, L2-d: L2-derived cell, M: Microsporocyte, ML: Middle Layer, MMC: Male Meiotic Cell, MP: Mature Pollen, Ms: Microspore, OSPC & ISPC: Outer & Inner Parietal Cell, PPC: Primary Parietal Cell, SC: Sporogenous Cell, T: Tapetum (Tapetal Cell), and Td: Tetrad. Scale bars: 25 μm

To test what causes the failure of pollen formation in the *Sbtdr-1* mutant, we then examined anther cell differentiation via semi-thin sections (Huang et al. 2017; Smith et al. 2023; Xin et al. 2017). Compared with other cereal crops like rice and maize, as well as the model organism *Arabidopsis*, anther development in sorghum remains less extensively characterized morphologically (Chen et al. 2019; Cheng et al. 1979; Kelliher and Walbot 2011; Laza et al. 2022; Raghavan 1988; Sanders et al. 1999; Xin et al. 2017; Zhang et al. 2011); thus, we first defined 13 main developmental stages based on observations from semi-thin sections of BTx623 anthers. At stage 1, three distinct cell layers, Layer 1 (L1), L2, and L3, are visible, from which all anther cells originate (Fig. 4A) (Kelliher and Walbot 2011; Laza et al. 2022; Sanders et al. 1999; Xin et al. 2017). L1 gives rise to the epidermis, while L2 develops into most of the somatic anther wall cells as well as the reproductive cells. L3 contributes to the formation of vascular and connective tissues. At stage 2, L2 produces the L2-derived cells (L2-d) (Fig. 4B). At this stage, the anther begins forming four lobes, with connective cells and innermost vascular cells emerging at the center. At stage 3, the epidermal cells enlarge, and divisions of L2-d cells generate large central archesporial (Ar) cells and primary parietal cells (PPC) (Fig. 4C). At stage 4, PPCs divide to form outer and inner secondary parietal cells (OSPC and ISPC), while Ar cells differentiate into sporogenous cells (SC) (Fig. 4D). At stage 5, the OSPCs differentiate into the endothecium (En), and the ISPCs further divide, differentiating into the middle layer (ML) and tapetum (T), completing the anther wall cell differentiation (Fig. 4E). Microsporocytes (M or pollen mother cells) also complete their differentiation at this stage. At stage 6, meiosis occurs in the microsporocytes, which thus become cytologically isolated from one another following callose deposition, forming male meiotic cells (MMCs) (Fig. 4F). The tapetal layer becomes thinner. Stage 7 features the formation of dyads (D) through the first meiotic division (Fig. 4G). At stage 8, tetrads (Td) are formed following the second meiotic division (Fig. 4H). Concurrently, the tapetal cells condense. At stage 9, tetrads release individual microspores (Ms) as callose surrounding them degrades (Fig. 4I). The middle layer is not visible. At stage 10, the microspores enlarge and vacuolate. The degeneration of tapetal cells initiates (Fig. 4J, DT: Degenerating Tapetum or Tapetal cells). At stage 11, pollen wall thickening indicates ongoing pollen development (DP: Developing Pollen), and the tapetum has largely degenerated (Fig. 4K). At stage 12, remnants of the endothecium are still present, and the septum begins to break down (Fig. 4L). Mature pollen (MP) is formed. Finally, at stage 13, mature pollen is released through anther dehiscence, with the anther lobes visibly breaking apart (Fig. 4M).

**Fig. 5:**
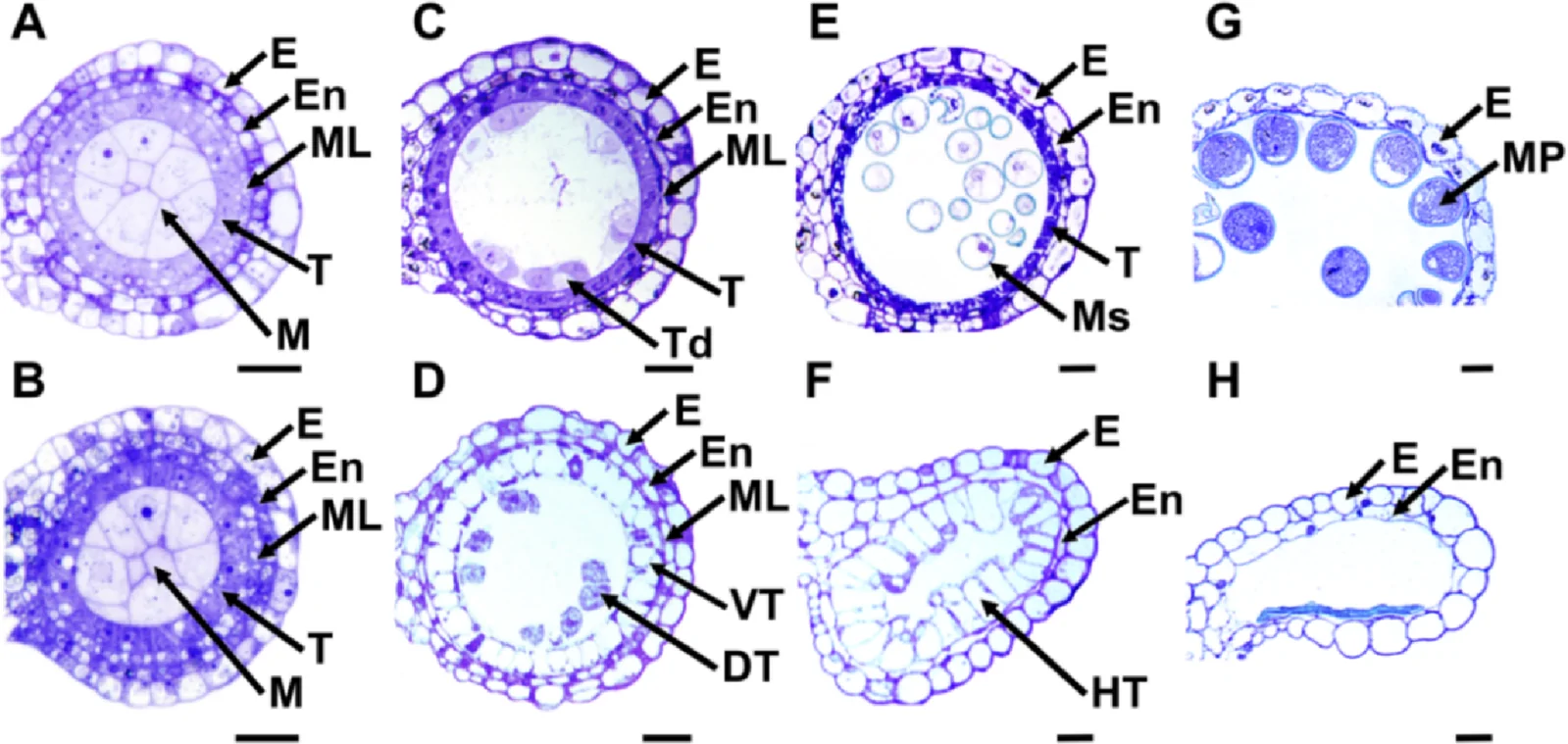
The *Sbtdr* mutant is defective in anther cell differentiation. (**A**, **B**) No detectable differences are observed between BTx623 (**A**) and *Sbtdr*-*1* (**B**) anthers at stage 5. (**C**, **D**) Compared to the BTx623 anther (**C**), the *Sbtdr*-*1* (**D**) anther showing abnormally vacuolated tapetal cells and degenerating tetrads (indicated by irregular cell membrane) at stage 8. (**E**, **F**) Tapetum and microspores are present in the BTx623 anther (**E**), while tapetal cells are hyper-vacuolated, crushing into the locule with no microspores (**F**) at stage 10. (**G**, **H**) The BTx623 anther (**G**) displaying mature pollen grains and absence of endothecium; in contrast, the *Sbtdr-1* anther (**H**) lacking pollen but showing remnants of undegenerated endothecium. DT: Degenerating Tetrad, E: Epidermis, En: Endothecium, HT: Hyper-vacuolated Tapetum (Tapetal Cell), M: Microsporocyte, ML: Middle Layer, MP: Mature Pollen, Ms: Microspore, T: Tapetum (Tapetal Cell), and Td: Tetrad, VT: Vacuolated Tapetum (Tapetal Cell). Scale bars: 25 μm

Compared to BTx623 anthers, *Sbtdr-1* anthers form all typical anther wall cell layers, including epidermis (E), endothecium (En), the middle layer (ML), tapetum (T), and the inner microsporocytes (M, Fig. 5A, B). No obvious abnormalities were detected before the tetrad stage. The first clearly visible defects in *Sbtdr-1* anthers appear at stage 8. In the BTx623 anther, tetrads (Td) are formed, and tapetal cells exhibit a condensed, less-vacuolated appearance (Fig. 5C). In contrast, the *Sbtdr-1* anther contains vacuolated tapetal cells (VT) at the same stage (Fig. 5D). The tetrads seem to be degenerating, indicated by their irregular cell membranes. As development progresses, microspores (Ms) are present in the BTx623 anther, and tapetal cells remain strongly stained, although normal tapetal degeneration has been initiated (Fig. 5E). In the *Sbtdr-1* anther, however, the tapetal cells become hyper-vacuolated and collapse inward toward the locule (HT: Hyper-vacuolated Tapetum or Tapetal Cell), where no microspores were observed (Fig. 5F). By stage 13, mature pollen (MP) grains have formed in the BTx623 anther (Fig. 5G). Conversely, the *Sbtdr-1* anther is essentially empty (Fig. 5H). The endothecium persists, indicating a failure of anther dehiscence. Taken together, these results suggest that impaired timely degeneration of tapetal cells lead to defective pollen development in *Sbtdr-1* anthers.

### The SbTDR gene encodes a bHLH transcription factor

**Fig. 6:**
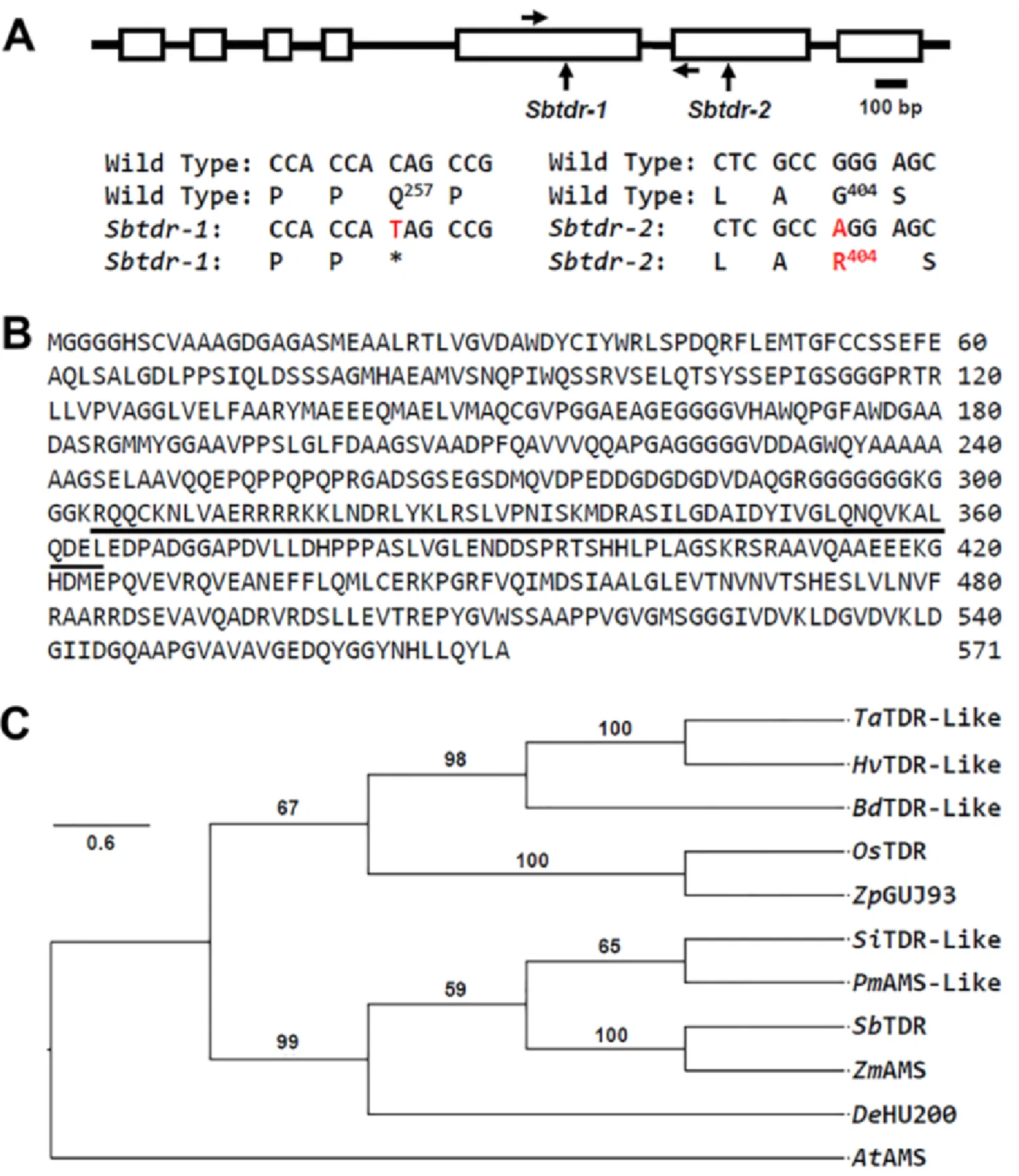
The SbTDR gene encodes a bHLH transcription factor. (**A**) The SbTDR gene consists of seven exons (shown as open boxes) and six introns. RT-PCR primer locations are indicated by black arrows. Mutation sites in the Sbtdr alleles are marked by arrows in exons 5 and 6. Scale bar: 100 bp. In Sbtdr-1, a C-to-T mutation introduces a premature stop codon (TAG) at Q^257^ in exon 5. In Sbtdr-2, a G-to-A mutation in exon 6 leads to a G^404^ to R^404^ substitution. (**B**) The SbTDR protein sequence. The putative bHLH domain is underlined. (**C**) Phylogenetic analysis of SbTDR-related proteins. A maximum likelihood tree was constructed in IQ-TREE using TDR protein sequences retrieved from UniProt. The SbTDR protein was used as the reference, and orthologues from representative monocot and dicot species were included for comparison. Bootstrap support values (%) are shown at branches. The Arabidopsis thaliana AMS sequence was used as the dicot outgroup to root the tree. Zm: Zea mays, Ta: Triticum aestivum, Hv: Hordeum vulgare, Bd: Brachypodium distachyon, Os: Oryza sativa, Zp: Zizania palustris, Si: Setaria italica, Pm: Panicum miliaceum, De: Digitaria exilis, and At: Arabidopsis thaliana

Screening of our sorghum mutant library (Jiao et al. 2016, 2017; Xin et al. 2008) identified two *Sbtdr* mutants, each carrying a single nucleotide mutation in the same gene, Sorbi_3004G017500. In *Sbtdr-1*, a C-to-T mutation introduces a premature stop codon (TAG) at amino acid position Q (glutamine)^257^ in exon 5, the gene’s largest exon (Fig. 6A). In *Sbtdr-2*, a G-to-A mutation in exon 6 results in a substitution of glycine (G) to arginine (R) at position 404 (G^404^ to R^404^). These mutations were further confirmed by sequencing PCR-amplified gene fragments of Sb04g001650 from each *Sbtdr* mutant.

The Sorbi_3004G017500 gene encodes a putative basic helix-loop-helix (bHLH) transcription factor (Fig. 6B), which is the orthologue of rice TAPETUM DEGENERATION RETARDATION (TDR) (Li et al. 2006; Niu et al. 2013) and *Arabidopsis* ABORTED MICROSPORES (AMS) (Sorensen et al. 2003). Accordingly, we designated these mutants as *Sbtdr* mutants. To investigate the relationship between *Sb*TDR and its orthologues, we performed a BLAST search using the full-length amino acid sequence of *Sb*TDR, and then conducted a phylogenetic analysis with orthologues in *Zea mays*, *Triticum aestivum*, *Hordeum vulgare*, *Brachypodium distachyon*, *Oryza sativa*, *Zizania palustris*, *Setaria italica*, *Panicum miliaceum*, *Digitaria exilis*, and *Arabidopsis thaliana* (Kearse et al. 2012; Nguyen et al. 2015). Our analysis revealed that *Sb*TDR and *Zea mays* AMS (or bHLH51) (Nan et al. 2022) were supported as an orthologous pair (Fig. 6C). Sequence comparisons showed that *Sb*TDR shares 82.4% of overall identity with *Zea mays* AMS and 57.6% with *Oryza sativa* TDR (Fig. S1).

### The SbTDR gene is primarily expressed in the tapetum

**Fig. 7:**
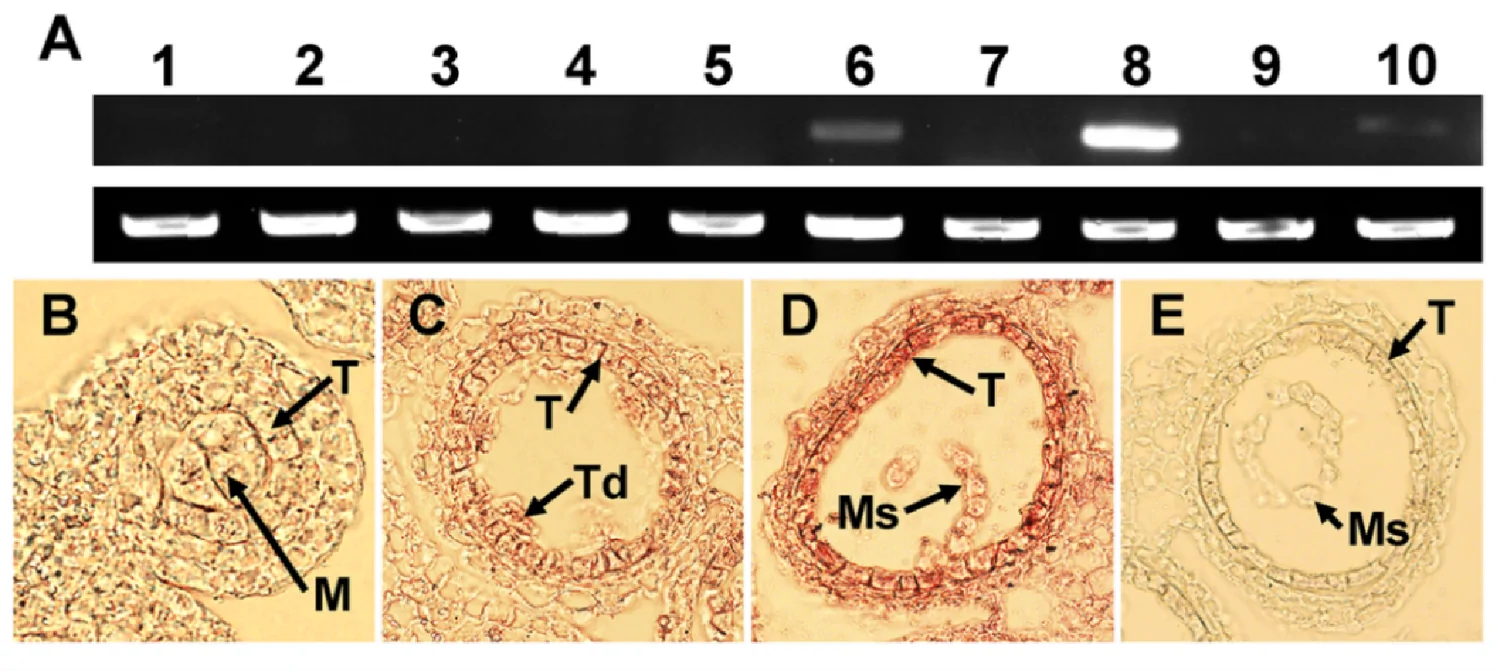
The *SbTDR* gene is predominantly expressed in tapetal cells. (**A**) RT-PCR result showing *SbTDR* is predominantly expressed in anthers containing microspores. Top panel: *SbTDR*. Bottom panel *ACTIN1* (Sb05g003880). Lane 1: Seedlings, Lane 2: Spikelets with anthers prior to the microsporocyte stage (pre-stage 5), Lane 3: Spikelets with anthers at the microsporocyte stage (stages 5 and 6), Lane 4: Anthers at the microsporocyte stage (stages 5 and 6), Lane 5: Spikelets with anthers at the tetrad stage (stage 8), Lane 6: Anthers at the tetrad stage (stage 8), Lane 7: Spikelets with anthers at the microspore stage (stages 9 and 10), Lane 8: Anthers at the microspore stage (stages 9 and 10), Lane 9: Spikelets with anthers at the pollen stage (at and after stage 11), and Lane 10: Anthers at the pollen stage (at and after stage 11). (**B**–**E**) RNA in situ hybridization results showing *SbTDR* spatial expression in anthers. (**B**) No expression at stage 5 (pre-meiosis, b). (**C**,** D**) A weak and strong expression in tapetum of anthers at the tetrad stage (stage 8, C) and the microspore stage (stages 9 and 10, D), respectively. (**E**) Negative control (sense probe) at the microspore stage (stages 9 and 10, D). Scale bars: 25 μm

To examine how the *SbTDR* gene controls anther development, we performed RT-PCR and RNA in situ hybridization to determine its spatial and temporal expression patterns. In sorghum, each sessile flower produces three anthers that develop synchronously. To stage fresh anthers, we squashed one anther in the 0.05% toluidine blue solution and then determined the anther stage by observing the released contents (microsporocytes, tetrads, microscopes or developing pollen) under a compound microscope. The remaining two anthers were collected for total RNA extraction. Our RT-PCR results show that *SbTDR* was not expressed in anthers at the microsporocyte stage (Fig. 7A, Lane 4), began to be weakly expressed in anthers at the tetrad stage (Fig. 7A, Lane 6), and became strongly expressed in anthers containing microspores (Fig. 7A, Lane 8). However, expression of *SbTDR* was barely detectable in anthers containing mature pollen (Fig. 7A, Lane 10) and absent in seedlings (Fig. 7A, Lane 1). The *SbTDR* expression was not observed in whole spikelets at any stage. These findings indicate that the *SbTDR* expression is both stage- and cell-type specific.

RNA in situ hybridization did not detect the *SbTDR* expression at stage 5, when the tapetal cell differentiation was complete and microsporocytes remained physically connected to one another within the locule (Fig. 7B, E). The expression of *SbTDR* was observed in the tapetum at the tetrad stage (stage 8, Fig. 7C), and *SbTDR* became strongly expressed in the tapetum by the microspore stage (stages 9 and 10, Fig. 7D). These findings indicate that *SbTDR* is anther-specific and predominantly expressed in the tapetum. The lack of *SbTDR* expression in stage-5 anthers when tapetal cell differentiation is complete suggests that *SbTDR* plays a more critical role in tapetal cell maintenance rather than the initiation of tapetal cell identity.

## Discussion

### The SbTDR gene plays a critical role in tapetum development

Anthers are vital for plant reproduction and play an essential role in agriculture, particularly in hybrid seed production, which often relies on male sterility. The tapetum, the innermost cell layer of the anther wall, is crucial for pollen development, as lack of the tapetum or the abnormal tapetum impedes microspore and pollen development, resulting in male sterility (Goldberg et al. 1993; Walbot and Egger 2016; Zhao 2009). Moreover, abiotic stress can impair tapetum function, reducing male fertility and leading to significant yield loss in crops (Begcy et al. 2019; Chaturvedi et al. 2021; De Storme and Geelen 2014; Malik and Zhao 2022; Parish et al. 2012; Smith et al. 2023; Smith and Zhao 2016). Our study demonstrates that the *SbTDR* gene is required for pollen development by controlling tapetal cell degeneration in sorghum. This work advances our understanding of the molecular mechanisms underlying anther cell differentiation in sorghum, a complex process for which only two regulatory genes have been identified to date (Chen et al. 2019; Xin et al. 2017; Zhao and Xin 2024).

Mutations in the *SbTDR* orthologous gene, *TDR* in rice (Li et al. 2006; Niu et al. 2013) and *AMS* in *Arabidopsis* (Sorensen et al. 2003), cause similar anther phenotypes, supporting the evolutionary conservation of key regulators of tapetum development. Similar to rice *TDR*, the expression of *SbTDR* is anther-specific, with moderate levels in the tapetum at the tetrad stage and stronger expression at the microspore stage. In a related study, we characterized the sorghum *ms8* mutant, which has a similar phenotype to that of *Sbtdr* (Xin et al. 2017). The *MS8* gene also encodes a bHLH transcription factor that is an orthologue of rice EAT1/DTD/bHLH141 (Ji et al. 2013; Niu et al. 2013; Zhao and Xin 2024). Notably, *MS8*, *EAT1*/*DTD*/*bHLH141*, rice *TDR*, and *SbTDR*, are all highly expressed in tapetal cells at the microspore stage, when tapetal cells are fully functional in supporting pollen development (Ji et al. 2013; Li et al. 2006; Niu et al. 2013; Zhao and Xin 2024). In rice, TDR forms a heterodimer with bHLH142 to activate the expression of *EAT1* by binding to its promoter, while EAT1 in turn upregulates expression of *AP25* and *AP37*, which induces tapetal programmed cell death (Ko et al. 2014; Niu et al. 2013). Although this remains to be tested, we hypothesize that SbTDR possibly controls tapetal programmed cell death through directly regulating the expression of *MS8* in sorghum.

Although loss-of-function mutations in TDR cause defective tapetum degeneration and male sterility, the gain-of-function of TDR remains unclear. Arabidopsis plants expressing a truncated AMS cDNA under the constitutive 35 S promoter exhibited abnormal anther development and reduced fertility (Sorensen et al. 2003). However, because the transgene encoded a truncated AMS protein, the observed phenotype likely resulted from interference with the endogenous AMS function rather than a true gain-of-function or ectopic expression of AMS.

### The anther development in Sorghum is similar to that of other cereal grains

In this study, we established a comprehensive anther developmental series for accurately staging sorghum anthers. Because the primary goal of this study was to characterize anther developmental defects in sorghum male sterile mutants using semi-thin sections, we adopted a simplified 13-stage cytological classification based on major cellular landmarks. This approach differs from the more detailed 18-stage morphological framework, which was mainly established from morphological and structural features observed by Cryo-VP-SEM throughout the entire course of anther development, from stamen primordium initiation to anther senescence (Laza et al. 2022). Our semi-thin section analysis of wild-type anthers reveals that anther cell differentiation in sorghum generally resembles that of rice and maize (Cheng et al. 1979; Kelliher and Walbot 2011; Raghavan 1988; Zhang et al. 2011). Unlike *Arabidopsis* (Ma 2005; Sanders et al. 1999; Zhao et al. 2002), male meiosis in sorghum leads to the production of dyads and tetrads, followed by the release of microspores. After microspore formation, we observed a few differences between sorghum and rice anther development. In rice, microspores accumulate starch and other reserves and gradually become round as they eventually develop into mature pollen. This process gives rise to a characteristic hook-shaped cell identified as bicellular pollen. However, in our sorghum samples, such hook-shaped cells were rare and likely resulted from processing artifacts rather than representing a true developmental stage, consistent with prior observations (Zhang et al. 2011). Additionally, in sorghum, the septum between locules appears to degrade earlier than in rice. Some late-stage samples also exhibited shrunken epidermal cells, likely due to an increased possibility of sample processing errors in late stages of anther development (Li et al. 2011; Zhang et al. 2011). The extended dehydration and infiltration steps used in our protocol may have improved tissue preservation, yielding better section quality, more rounded cells at the microspore-to-bicellular pollen transition, and more intact anther wall structures at later stages.

### Potential application of Sbtdr in hybrid sorghum breeding

Male sterility is widely used in crop hybrid breeding through either cytoplasmic male sterility (CMS)-based three-line systems or nuclear male sterility (NMS)-based two-line systems (Chang et al. 2016; Wu et al. 2016; Xin et al. 2017; Zhang et al. 2013; Zhou et al. 2014). Currently, CMS is the only system employed for hybrid breeding in sorghum (Jordan et al. 2011; Praveen et al. 2015; Rooney 2004; Xin et al. 2017; Zhao and Xin 2024). This CMS system requires a male sterile A line, a maintainer B line, and a restorer R line. Although effective, this system is complex, costly, and restricts hybrid combinations to specific A/B and R line combinations, limiting the use of diverse germplasm resources and the exploitation of hybrid vigor. For example, most commercial hybrids rely on the A1 cytoplasm, raising concerns about vulnerability to pests and diseases, as exemplified by the Southern Corn Leaf Blight epidemic (Ullstrup 1972).

In contrast, two-line systems are simpler and have been employed in crops such as rice, where Photoperiod/Thermo-sensitive Genic Male Sterile (PTGMS) lines can be used for hybrid seed production under restrictive conditions and propagated under permissive conditions (Chang et al. 2016; Wu et al. 2016; Xin et al. 2017; Zhang et al. 2013; Zhou et al. 2014). However, these conditional systems are environmentally unstable and are not available in many crops, including sorghum, preventing the development of two-line hybrid breeding in all crops.

Recently, non-conditional NMS mutants and their causal genes were genetically engineered for developing two-line hybrid breeding systems in maize and rice (Chang et al. 2016; Wan et al. 2019; Wu et al. 2016; Zhang et al. 2018). As sorghum becomes increasingly important as a grain, forage, sweet, and bioenergy crop (Chen et al. 2019; de Siqueira Ferreira et al. 2013; Morris et al. 2013; Paterson et al. 2009; Rhodes et al. 2014; Rooney et al. 2007; Smith et al. 2023; Upadhyaya et al. 2009; Xin et al. 2012, 2017; Zhao and Xin 2024), similar systems could substantially expand breeding opportunities. Our study shows that the *Sbtdr* mutant exhibits complete male sterility without obvious defects in vegetative or floral organs other than the anther and pollen. Therefore, the *Sbtdr* mutant and its causal gene *SbTDR* represent valuable genetic resources for the future development of NMS-based two-line hybrid breeding systems in sorghum.

## Supplementary Information

Below is the link to the electronic supplementary material.

Supplementary Material 1

## Data Availability

All original data files will be made available upon request.
