# Supplementary material for: The SbTDR gene is required for anther development and male fertility in Sorghum bicolor
Source: Plant Reprod. 2026 Aug 25;39(3-4):12. doi: 10.1007/s00497-026-00546-4 (PMC13506635; doi:10.1007/s00497-026-00546-4)
Supplement: Supplementary file 1 [file 497_2026_546_MOESM1_ESM.docx]

**Supporting Information**

**Fig. S1. Sequence alignment of the *Sb*TDR protein and its orthologs.** The black box indicates the bHLH domain. *Zm*: *Zea mays*, *Ta*: *Triticum aestivum*, *Hv*: *Hordeum vulgare*, *Bd*: *Brachypodium distachyon*, *Os*: *Oryza sativa*, *Zp*: *Zizania palustris*, *Si*: *Setaria italica*, *Pm*: *Panicum miliaceum*, *De*: *Digitaria exilis*, and *At*: *Arabidopsis thaliana*.

| **Table S1. Primers used in this study** | | |
| --- | --- | --- |
| Primer Name | Sequence | Purpose |
| Sorbi_3004G017500 Seq/RT F | GACCCGTTCCAGGCGGTG | *SbTDR* sequencing |
| Sorbi_3004G017500 Seq R | ACGGCAGCGCGGAAGACG | *SbTDR* sequencing |
| Sb05g003880 RT F | TTCACGAGACTACCTACAAC | *ACTIN1* RT-PCR control |
| Sb05g003880 RT R | ATCAAGTGAGAAGCATAGGT | *ACTIN1* RT-PCR control |
| Sorbi_3004G017500 Seq/RT F | GACCCGTTCCAGGCGGTG | *SbTDR* RT-PCR |
| Sorbi_3004G017500 RT R  Sorbi_3004G017500 IS F | GTTCTGCAGACCCACGATGT  GTGTGGACGTGAAGCTCGAT | *SbTDR* RT-PCR  in situ hybridization |
| Sorbi_3004G017500 IS R | TCCACACTCATTTGAACCAACAC | in situ hybridization |
